# Judicialization and cancer: quality of life of patients and caregivers in the COVID-19 pandemic

**DOI:** 10.1186/s12955-023-02173-3

**Published:** 2023-08-11

**Authors:** Leila Abou Salha, José Elmo de Menezes, Danilo Rocha Dias, Virginia Visconde Brasil, Pedro Lopes Ferreira, Jolivê Mendes de Santana Filho, Maria Alves Barbosa

**Affiliations:** 1 Faculty of Medicine, Federal University of Goias, Goiânia, Goiás 74605-050 Brazil; 2 Federal Institute of Education, Science, and Technology of Goias, Goiânia, 74605-900 Goiás Brasil; 3 Faculty of Dentistry, Federal University of Minas Gerais, Belo Horizonte, 31270-901 Brazil; 4 Faculty of Nursing, Federal University of Goias, Goiânia, Goiás 74605-080 Brazil; 5 Faculty of Economics, University of Coimbra, 3004-512 Coimbra, Portugal; 6 Federal Institute of Goias, Goiânia, Goiás 74055-110 Brazil

**Keywords:** Quality of life, Antineoplastic agents, Cancer, Judicialization of health, Caregivers

## Abstract

**Background:**

In Brazil, cancer patients and caregivers of cancer patients seek judicial intervention for free access to medications from the public health system. Indeed, the COVID-19 pandemic potentially affected the health-related quality of life of cancer patients and caregivers of cancer patients. This study aimed to describe the sociodemographic profile and assess the health-related quality of life of patients and caregivers in the state of Goias, Brazil, in 2020.

**Methods:**

A cross-sectional study was conducted using the Medical Outcomes Study 36—Item Short Form Health Survey (SF-36) and a sociodemographic questionnaire.

**Results:**

A total of 88 (67,7%) patients and 42 (32,3%) caregivers participated in the study, mostly women (55,5%); aged from 18 to 60 (66%) years old; with up to nine years of education (73,1%) and monthly family income lower than the minimum wage (69,2%); married or in a stable union (92,3%); living with multiple people in the same household (73,8%). The quality of life domains with the best scores were mental health for patients and pain for caregivers. The most affected quality of life domain was physical limitation for patients and caregivers. Factors associated with better quality of life were female gender and age between 18 and 60 years in patients, more than 9 years of education, living with multiple people in the same house, and having a monthly family income higher than US$200 for caregivers.

**Conclusion:**

The study found evidence of physical and emotional vulnerability during the pandemic, highlighting the need to strengthen public policies of assistance support to this population.

## Introduction

Since the beginning of 2020, the novel coronavirus (COVID-19) brought isolation [1, 2], the recognition of a global pandemic [3], and transmission in households and hospitals [4], and there is still much to learn about the susceptibility of groups of individuals with comorbidities.

The increased risk of non-transmissible chronic diseases, such as cancer, may be related to immunosuppression resulting from antineoplastic treatment and tumor malignancy [5]. However, cancer treatments and surgeries were suspended due to the pandemic [6–8], and treatment initiation was postponed, with possible future effects on cancer mortality [9, 10].

The success of oncological treatment is primarily determined by early diagnosis and adequate treatment [11]. However, the public health infrastructure responsible for cancer care does not promote quick access to cancer medications, mainly due to the high cost of treatment, generating inequities in access to treatment at individual and social levels [12, 13].

Meanwhile, cancer is a global health problem. In 2020, there were an estimated 19,292,789 new cases in both sexes and almost 10 million deaths from cancer worldwide [14]. By 2040, it is estimated that there will be 28.4 million new cases [15].

Indeed, in Brazil, in 2020, there were 592,212 new cases of cancer, the most prevalent, excluding non-melanoma skin cancers, prostate, breast, colorectal, lung, and thyroid cancer [16], of this total, 20,000 new cases in the state of Goias [17, 18].

The burden of cancer incidence and mortality is growing rapidly worldwide as a reflection of aging and population growth, combined with changes in the prevalence and distribution of the primary risk factors. Risk factors can be environmental, genetic, or rooted in behaviors, habits, or customs typical of a particular social and cultural environment [19].

However, despite being a significant cause of morbidity and mortality worldwide and a barrier to increasing life expectancy, the incidence is independent of the level of human development [16].

Quality of life is an individual subjective perception of significant life aspects that can be used to evaluate treatment effects in cancer patients. It allows physical health, emotional health, social relationships, environmental context, and spirituality to be assessed as a predictor of morbidity and mortality in cancer [20, 21].

In Brazil, health is a constitutional right, so public policies are put in place that guarantee access to medications [22]. Brazil constitutionally recognises full and universal access to medicines as part of the right to health, with a view to health with quality of life, and adopts public policies to guarantee it [22, 23].

In cancer, public access to treatment is carried out in 317 centers or units of high complexity in oncology enabled by the Ministry of Health, distributed throughout the Brazilian territory, to standardize and acquire the medicines and promote the appropriate treatment [14, 19].

Despite this, since the beginning of the unified health system (*SUS,* Brazilian national public health), the Brazilian population has used the judicial system to secure this right and gain access to the medications they need. This phenomenon is called the judicialization of pharmaceutical care and began with demands for antiretrovirals in the 1990s [24].

The judicialization of pharmaceutical assistance related to antineoplastic drugs has increased significantly in recent years and represents an important mechanism for access to medicines in the SUS [25, 26].

The judicialization reverses the organization of the system and changes its functioning in the provision of care, because it highlights that there are failures in public access to comprehensive and equitable assistance provided in the Federal Constitution [25, 26].

Cancer is recognized as a family disease due to the structural, socioeconomic, and emotional changes caused by the disease that affect all individuals in the family nucleus, and health care should be extended in this context [27, 28].

Cancer patients resort to the judiciary to request medication for various reasons, such as when a medication is not registered with the National Health Surveillance Agency (*ANVISA*), the medication is not described in the Clinical Protocols and Therapeutic Guidelines (PCDT) as being a therapy of choice, or even when standard front-line drugs are out of stock in the public service [29, 30]. In addition, caregivers can request judicial intervention on behalf of the individual requesting medication, an elderly patient, a child, or a patient with a disability or a rare disease [31].

Judicial intervention in *SUS* managerial activities impacts individual patients by enabling access to medications for prescribed treatments and the possibility of a better prognosis for cancer. It also has social impacts, promoting an imbalance in public health expenditures. The judicialization phenomenon involves political, social, ethical, and health aspects, which go far beyond its legal component and the management of public services [32].

This study aimed to describe the sociodemographic profile and evaluate the HRQoL of cancer patients and informal or family caregivers of cancer patients during the COVID-19 pandemic who requested medication through administrative and judicial channels in the state of Goias—Brazil in 2020.

## Methods

### Study design

A cross-sectional study was carried out from March 2020 to March 2021 at a state reference center for the supply of high-cost medicines (CEMAC) in the state of Goias, Brazil.

Of the 377 total individuals (patients or caregivers) registered at CEMAC in 2020 to receive cancer drugs, all patients and caregivers aged 18 years or older who received antineoplastic drugs were included regardless of the type of cancer, stage of the disease, and amount of medication received. Registered patients who had their treatment medically suspended and registered caregivers with no direct link with the individual with cancer (medical transport driver, health unit secretary, lawyer) were excluded.

**Fig. 1:**
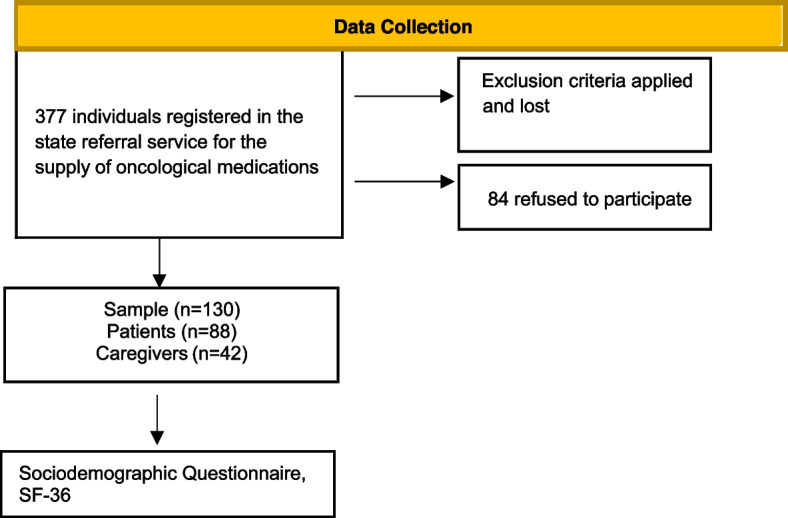
Study data collection flowchart

The intentional sample consisted of 130 participants, 88 patients, and 42 caregivers, according to the flowchart (Fig. 1).

### The instruments

For data collection, two questionnaires were used, one sociodemographic and the other assessing quality of life, the Medical Outcomes Study 36—Item Short Form Health Survey (SF-36). These were sent electronically on WhatsApp (Meta Inc. Menlo Park, CA, USA) and by e-mail, along with the research consent and clarification form in three successive invitations (in March, June, and December 2020).

The sociodemographic questionnaire included the type of respondent (individual with cancer or caregiver), age, sex, education, living in a household with multiple people, family income, religion, marital status, and the route of requesting the medication (administrative or judicial). In addition, the Brazilian version of the SF-36 instrument was used to assess the outcome variable (quality of life) [33].

The SF-36 was developed in 1992 by Ware and Sherbourne and validated in Brazil by Ciconelli et al. [33, 34]. The Portuguese version was chosen because it is validated and reproducible in the socioeconomic and cultural conditions of the Brazilian population. It is recognized as an instrument to assess quality of life, used in population surveys, health policy studies, and health condition assessment studies [34–39].

The instrument has 36 items forming 8 domains: Functional Capacity (FC) with 10 items, Physical Aspects (PA) with 4 items, pain with 2 items, General Health Status (GHS) with 5 items, Vitality (VIT) with 4 items, Social Aspects (SA) with 2 items, Emotional Aspects (EA) with 3 items, Mental Health (MH) with 5 items and a comparative assessment question between current health conditions and that of a year earlier. Physical health includes the first four domains (FC, PA, Pain, and GHS), and mental health the last four (VIT, SA, EA, and MH).

The scores obtained are calculated using pre-established norms, and the values of the questions are transformed into scores for eight domains from 0–100. Each domain is analyzed separately. The instrument also includes a non-scored question (question no. 2) that aims to compare health changes that occurred in one year [33].

By combining the scores of each dimension, the SF-36 allows 2 integrated components to be calculated: the physical health component (PHC) and the mental health component (MHC) [32]. The PHC comprises the following dimensions: functional capacity (performing daily activities, such as the ability to take care of oneself, get dressed, shower, and climb stairs); physical aspects (impact of physical health on the performance of daily or professional activities); pain (pain level and impact on performing daily or professional activities); general health status (subjective perception of general health status). The MHC comprises the following dimensions: vitality (subjective perception of health status); social aspects (reflection of physical health condition on social activities); emotional aspects (reflection of emotional conditions on performing daily or professional activities); and mental health (mood and well-being scale).

The SF-36 presents a final score from zero to one hundred (0–100), where zero corresponds to the worst general health status and one hundred to the best health status. The survey questions were structured in scales with different scoring possibilities (1 to 6; 1 to 5; 1 and 2; 1, 2, and 3). The intensity can increase or decrease according to the question.

### Statistical analysis

Microsoft® Excel 2019 program was used for data tabulation. The statistical program adopted for data analysis was SPSS version 28.0.1 [SPSS Inc., Chicago, IL, USA] for Windows.

The normality of the data was obtained parametrically by the bootstrap method [40]. The method allows resampling of the original sample with replacement, and, for each resampling, the estimate of interest is calculated [41]. In this study, the resample was 1000. In addition, Shapiro–Wilk and Kolmogorov–Smirnov tests were performed to calculate normality.

Cronbach's Alpha internal consistency test was used to test the survey's reliability. For the descriptive analysis of the profile data, respondents (patient and caregiver, total and by group), means, and standard deviations were calculated. The variables evaluated were sex, age, education level, marital status, other household members living with the patient, family income, and religion.

Pearson's correlation was used to compare the SF-36 domains, considering *r* = 1 as perfect, *r* > 0.7 strong, *r* > 0.5 moderate, *r* < 0.5 weak, and *r* = 0 as no correlation. The significance level adopted in the study was 5%.

Logistic regression was performed to control the confounding variables between the independent variables and the outcome (generalized linear model), considering statistical significance (*p* < 0.05).

The research involved human beings and complied with the ethical and legal precepts regulated by the National Health Council according to Resolutions No. 466/2012 and No. 510/2016 and was submitted to the Research Ethics Committee of the Federal University of Goias, decision no. 2.831.905 – CAAE 93238318.7.0000.5083 [42, 43].

## Results

### Demographical characteristics of participants

The majority of participants were women, 18–60 years old, with up to 9 years of education, income up to 1 × the minimum wage (in 2020, which was R$1.045,00 or about USD$200 per month), married or in a stable relationship, who live with more people in the same household and declared having a religion. Access occurred through individual lawsuits. In addition, informal or family caregivers participated in the study (Table 1).

**Table 1:** Sociodemographic profile of the study

VARIABLES	Patient *n* = 88	Caregiver *n* = 42
Gender		
Male	38 (29.2%)	19 (14.6%)
Female	50 (38.5%)	23 (17%)
Age		
18–60	58 (44.6%)	28 (21.5%)
over 60	30 (23.1%)	14 (10.8%)
Years of study		
up to 9 years	69 (53.1%)	26 (20%)
over 9 years	19 (14.6%)	16 (12.3%)
Marriage status		
with partner	64 (67.7%)	32 (24.6%)
no partner	24 (18.5%)	10 (7.7%)
Residents		
lives alone	24 (18.5%)	10 (7.7%)
lives with others	64 (49.2%)	32 (24.6%)
Family income		
up to 1 × MW	64 (49.2%)	26 (20%)
above 1 × MW	24 (18.5%)	16 (12.3%)
Declared religion		
Yes	71 (54.6%)	26 (20%)
No	17 (13.1%)	16 (12.3%)

*Legend*: *MW* Minimum wage in 2020, which was R$1.045,00 or about USD$200 per month [44]

#### Health-related quality of life

The domains of the outcome variable (quality of life) were analyzed using the SF- 36 Health Survey scores, the mean, and the respective standard deviation. It was observed that the domains with the highest means were pain (56.64, SD 7.60) and mental health (56.12, SD 2.92), and the most affected domain was physical limitation (mean 39.20, SD 7.32), both among patients and caregivers. Again, there was no significant difference between the type of respondent, with caregivers having higher scores in all domains than patients. However, it is notable that none of the mean scores ranging from 0–100 practically exceeded 60 (Table 2).

**Table 2:** Short form health survey of the patients and caregivers studied

Domains	Patients		Caregivers	
	Mean	Standard Deviation	Mean	Standard Deviation
Functional capacity-D1	42.43	3.11	52.72	4.72
Physical limitation-D2	34.87	4.99	43.53	6.71
Pain-D3	50.04	3.03	63.25	4.33
General health status-D4	43.04	1.88	53.07	2.85
Vitality-D5	46.78	2.10	55.35	2.70
Social aspects-D6	54.23	3.07	58.63	4.95
Emotional aspects-D7	38.09	5.09	51.79	7.37
Mental health-D8	55.11	2.25	57.14	3.17

#### Correlation between scores of SF-36 instruments

In the correlation between the SF-36 domains, for the patient group, there was a statistically significant mild to moderate positive correlation among all the SF-36 domains, except functional capacity and mental health.

The synthesized components showed a moderate to very strong positive correlation with the SF-36 domains and a very strong correlation with each other. The Physical Health Component correlated with the Pain, Physical Capacity, and Physical.

Limitations domains, while the Mental Health Component presented a very strong correlation with the Physical Limitations, Pain, Emotional Aspects, and Mental Health domains (Table 3).

**Table 3:** Pearson's correlation between the health-related quality of life domains and synthesized components of the SF-36 for cancer patients

	Functional capacity	Physical aspects	Pain	General health status	Vitality	Social aspects	Emotional aspects	Mental health	PHC	MHC
Functional capacity	*r* = 1	*r* = 0,60	*r* = 0,69	*r* = 0,52	*r* = 0,52	*r* = 0,47	*r* = 0,38	*r* = 0,28	*r* = 0,85	*r* = 0,53
		*p* < 0,001	*p* < 0,001	*p* < 0,001	*p* < 0,001	*p* < 0,001	*p* < 0,001	*p* = 0,008	*p* < 0,001	*p* < 0,001
Physical aspects	*r* = 0,60	*r* = 1	*r* = 0,60	*r* = 0,40	*r* = 0,42	*r* = 0,53	*r* = 0,72	*r* = 0,34	*r* = 0,86	*r* = 0,71
	*p* < 0,001		*p* < 0,001	*p* < 0,001	*p* < 0,001	*p* < 0,001	*p* < 0,001	*p* < 0,001	*p* < 0,001	*p* < 0,001
Pain	*r* = 0,69	*r* = 0,60	*r* = 1	*r* = 0,50	*r* = 0,54	*r* = 0,48	*r* = 0,49	*r* = 0,39	*r* = 0,85	*r* = 0,62
	*p* < 0,001	*p* < 0,001		*r* = 1	*p* < 0,001	*p* < 0,001	*p* < 0,001	*p* < 0,001	*p* < 0,001	*p* < 0,001
General health status	*r* = 0,52	*r* = 0,40	*r* = 0,50	*r* = 1	*r* = 0,33	*r* = 0,30	*r* = 0,29	*r* = 0,35	*r* = 0,66	*r* = 0,41
	*p* < 0,001	*p* < 0,001	*p* < 0,001		*p* = 0,002	*p* = 0,004	*p* = 0,008	*p* < 0,001	*p* < 0,001	*p* < 0,001
Vitality	*r* = 0,52	*r* = 0,42	*r* = 0,54	*r* = 0,33	*r* = 1	*r* = 0,56	*r* = 0,36	*r* = 0,51	*r* = 0,56	*r* = 0,72
	*p* < 0,001	*p* < 0,001	*p* < 0,001	*p* = 0,002		*p* < 0,001	*p* < 0,001	*p* < 0,001	*p* < 0,001	*p* < 0,001
Social aspects	*r* = 0,47	*r* = 0,53	*r* = 0,48	*r* = 0,30	*r *= 0,56	*r* = 1	*r* = 0,41	*r* = 0,32	*r* = 0,56	*r* = 0,74
	*p* < 0,001	*p* < 0,001	*p* < 0,001	*p* = 0,004	*p* < 0,001		*p* < 0,001	*p* = 0,002	*p* < 0,001	*p* < 0,001
Emotional aspects	*r* = 0,38	*r* = 0,72	*r* = 0,49	*r* = 0,29	*r* = 0,36	*r* = 0,41	*r* = 1	*r* = 0,51	*r* = 0,62	*r* = 0,84
	*p* < 0,001	*p* < 0,001	*p* < 0,001	*p* = 0,008	*p* < 0,001	*p* < 0,001		*p* < 0,001	*p* < 0,001	*p* < 0,001
Mental health	*r* = 0,28	*r* = 0,34	*r* = 0,39	*r* = 0,35	*r* = 0,51	*r* = 0,32	*r* = 0,51	*r* = 1	*r* = 0,41	*r* = 0,72
	*p* = 0,008	*p* < 0,001	*p* < 0,001	*p* < 0,001	*p* < 0,001	*p* = 0,002	*p* < 0,001		*p* < 0,001	*p* < 0,001
PHC	*r* = 0,85	*r* = 0,86	*r* = 0,85	*r* = 0,66	*r *= 0,56	*r* = 0,56	*r* = 0,62	*r* = 0,41	*r* = 1	*r* = 0,72
	*p* < 0,001	*p* < 0,001	*p* < 0,001	*p* < 0,001	*p* < 0,001	*p* < 0,001	*p* < 0,001	*p* < 0,001		*p* < 0,001
MHC	*r* = 0,53	*r* = 0,71	*r* = 0,62	*r* = 0,41	*r* = 0,72	*r* = 0,74	*r* = 0,84	*r* = 0,72	*r* = 0,72	*r* = 1
	*p* < 0,001	*p* < 0,001	*p* < 0,001	*p* < 0,001	*p* < 0,001	*p* < 0,001	*p* < 0,001	*p* < 0,001	*p* < 0,001	

*PHC* physical health synthesized component, *MHC* mental health synthesized component

In the group of caregivers, moderately intense positive correlations were identified between the functional capacity and pain domains; physical limitation and vitality and emotional aspects; and between pain, social aspects, and emotional aspects (Table 4).

**Table 4:** Values obtained from Pearson's correlation between the health-related quality of life domains and synthesized components of the SF-36 for caregivers

	Functional capacity	Physical aspects	Pain	General health status	Vitality	Social aspects	Emotional aspects	Mental health	PHC	MHC
Functional capacity	*r* = 1	*r* = 0,30	*r* = 0,56	*r* = 0,26	*r* = 0,16	*r* = 0,25	*r* = 0,37	*r* = -0,032	*r* = 0,71	*r* = 0,28
		*p* = 0,05	*p* < 0,001	*p* = 0,09	*p* = 0,31	*p* = 0,09	*p* = 0,02	*p* = 0,8	*p* < 0,001	*p* = 0,07
Physical aspects	*r* = 0,30	*r* = 1	*r* = 0,32	*r* = 0,32	*r* = 0,48	*r* = 0,39	*r* = 0,66	*r* = 0,24	*r* = 0,78	*r* = 0,57
	*p* = 0,05		*p* = 0,04	*p* = 0,04	*p* = 0,001	*p* = 0,013	*p* < 0,001	*p* = 0,09	*p* < 0,001	*p* < 0,001
Pain	*r* = 0,56	*r* = 0,32	*r* = 1	*r* = 0,39	*r* = 0,41	*r* = 0,48	*r* = 0,39	*r* = 0,23	*r* = 0,74	*r* = 0,46
	*p* < 0,001	*p* = 0,04		*p* = 0,013	*p* = 0,007	*p* = 0,001	*p* = 0,013	*p* = 0,15	*p* < 0,001	*p* = 0,002
General health status	*r* = 0,26	*r* = 0,32	*r* = 0,39	*r* = 1	*r* = 0,43	*r* = 0,06	*r* = 0,33	*r* = 0,10	*r* = 0,60	*r* = 0,28
	*p* = 0,09	*p* = 0,04	*p* = 0,013		*p* < 0,001	*p* = 0,710	*p* = 0,04	*p* = 0,54	*p* < 0,001	*p* = 0,07
Vitality	*r* = 0,16	*r* = 0,48	*r* = 0,41	*r* = 0,43	*r* = 1	*r* = 0,62	*r* = 0,65	*r* = 0,68	*r* = 0,52	*r* = 0,84
	*p* = 0,31	*p* = 0,001	*p* = 0,007	*p* < 0,001		*p* < 0,001	*p* < 0,001	*p* < 0,001	*p* < 0,001	*p* < 0,001
Social aspects	*r* = 0,25	*r* = 0,39	*r* = 0,48	*r* = 0,06	*r* = 0,62	*r* = 1	*r* = 0,60	*r* = 0,49	*r* = 0,44	*r* = 0,83
	*p* = 0,09	*p* = 0,013	*p* = 0,001	*p* = 0,710	*p* < 0,001		*p* < 0,001	*p* < 0,001	*p* < 0,001	*p* < 0,001
Emotional aspects	*r* = 0,37	*r* = 0,66	*r* = 0,39	*r* = 0,33	*r* = 0,65	*r* = 0,60	*r* = 1	*r* = 0,48	*r* = 0,65	*r* = 0,89
	*p* = 0,02	*p* < 0,001	*p* = 0,013	*p* = 0,04	*p* < 0,001	*p* < 0,001		*p* < 0,001	*p* < 0,001	*p* < 0,001
Mental health	*r* = -0,032	*r* = 0,24	*r* = 0,23	*r* = 0,10	*r* = 0,68	*r* = 0,49	*r* = 0,48	*r* = 1	*r* = 0,20	*r* = 0,72
	*p* = 0,8	*p* = 0,09	*p* = 0,15	*p* = 0,54	*p* < 0,001	*p* < 0,001	*p* < 0,001		*p* = 0,19	*p* < 0,001
PHC	*r* = 0,71	*r* = 0,78	*r* = 0,74	*r* = 0,60	*r* = 0,52	*r* = 0,44	*r* = 0,65	*r* = 0,20	*r* = 1	*r* = 0,59
	*p* < 0,001	*p* < 0,001	*p* < 0,001	*p* < 0,001	*p* < 0,001	*p* < 0,001	*p* < 0,001	*p* = 0,19		*p* < 0,001
MHC	*r* = 0,28	*r* = 0,28	*r* = 0,46	*r* = 0,28	*r* = 0,84	*r* = 0,83	*r* = 0,89	*r* = 0,72	*r* = 0,59	*r* = 1
	*p* = 0,05	*p* = 0,07	*p* = 0,002	*p* = 0,07	*p* < 0,001	*p* < 0,001	*p* < 0,001	*p* < 0,001	*p* < 0,001	

*PHC* synthesized physical health component, *MHC* synthesized mental health component

The synthesized components showed a mild to very strong positive correlation with the SF-36 domains and a very strong positive correlation with each other. The physical health composite score showed the strongest correlations with the Pain and Emotional Aspects domains, while mental health showed the strongest correlations with the Vitality, Social Aspects, and Emotional Aspects domains (Table 4). However, comparing these variables does not suggest a cause-and-effect relationship between them, but it signals a direction and convergence of this association.

The generalized linear regression analysis results show no difference between the access routes (administrative and judicial). However, being between 18 and 60 was significantly different from being over 60. As well as concerning the number of residents in the household, living with more people was significant. In terms of income, it was significant to have an income above the minimum wage compared to those below the minimum wage concerning the Physical Component (Table 5).

**Table 5:** Factors potentially associated with the QoL of 88 patients with cancer, obtained from generalized linear regression analysis (*p* < 0.05), Goias, Brazil, 2020

VARIABLES	Mean SF 36	*P*-value	Reference category
	Coefficient		
Gender			
Male	1.927	0.048	Female
Age Range			
More than 60 years	2.238	0.00935	18–60 years

The factors potentially associated with a worse quality of life in patients are: being male and being over 60 years of age. Regarding the caregiver: having less than 9 years of education, living alone, and having a family income of up to 1 × MW (Table 6).

**Table 6:** Factors potentially associated with the QoL of 42 caregivers of individuals with cancer in the study, obtained from generalized linear regression analysis (*p* < 0.05), Goias, Brazil, 2020

VARIABLES	Mean SF 36	*P*-value	Reference Category
	Coefficient		
Years of study			
Up to 9 years	4.624	0.0127	More than 10 years
Residents			
Live alone	-10.870120	0.01075	Live with others
Family income			
Up to 1 × MW	-7.67011	0.01209	More than 1 MW

*MW* Minimum wage in 2020 was 1,045.00 reais or about USD$200 per month [44]

## Discussion

The profile of study participants, mostly adult women, with a partner, with income below the national minimum wage and fewer than nine years of education, is similar to other national and international studies with individuals with cancer and caregivers [45–50].

Women's high participation in health services has been frequently noted and may suggest more concern for self-care [51–59]. In addition, the role of caregivers is highlighted, as cancer is a family disease, and care is still an essentially female activity in Brazilian culture [60–63].

In most cases, a family member becomes a caregiver out of necessity. They are commonly wives or daughters who have trouble in formal jobs or are even forced to abandon them, adversely affecting the family's income, and overwork causes adverse effects on the caregiver's quality of life [64, 65]. This study's profile of adult women as informal caregivers is in line with other studies [66–69].

Men's involvement in care, especially for the elderly, has increased in recent years, although there is little research on informal care provided by male family members. A recent study reveals the strain and social isolation associated with male caregiving, as well as the gratitude and reciprocity that results [70].

Care as a profession with formal caregivers has been recognized since 2011 in the national register of occupations [71]; however, in this study, only family caregivers, also called informal caregivers, participated. These caregivers are not formally recognized professionals and do not have the rights typically associated with a formal job.

The economic classes are a group of people with similar cultural, political, and economic standards, which can be divided in descending order in relation to household income into A, B, C, D, E. Most participants reported an income of up to 1 × the minimum wage, R$1,045.00 (about USD$200) in 2020 [44], belonging to economic classes D and E [53]. This income corresponds to a lower level than the average real per capita income in the country [53], negatively impacting the quality of life of caregivers in the study and possibly reflecting the COVID-19 global pandemic on the income of this population.

COVID-19 greatly affected the economy, exacerbating unemployment and informality. In the last quarter of 2020, the official unemployment rate was 13.9% [71], causing an increase in informal work and demanding a state protection network with public policies to combat poverty [67, 68, 71, 72]. Even so, informality in the economy reached 40%, indicating economic instability and the reduction of formal jobs [67–71].

Along with reducing income, rising unemployment during the pandemic suggested many men were present in their homes helping to care for their family members with cancer. However, despite this, the division of tasks is still unequal, with care activities naturalized as female [60, 66, 73] aggravated by the pandemic that amplified inequality and reduced women's incomes, especially [73].

The predominant age group was between 18 and 60 years old, married or with a partner. Individuals over 60 years of age have fragile economic and labor situations, mostly in informal activities, with a sharp decrease in income and a lower socioeconomic level during the pandemic [73–75].

In 2019, in Brazil, more than 120,000 deaths from cancer were recorded in individuals aged 30 to 69 years. Furthermore, it is considered the leading cause of death from chronic non-communicable diseases among women as of 2014 [76].

The elderly is more susceptible to loneliness, reinforced by the social isolation imposed by the pandemic, with damage to physical and mental health [77, 78]. It is reinforced that loneliness is a significant predictor of mortality and functional capacity decline that needs to be evaluated as a significant risk factor [78, 79].

Regarding the quality of life domains, the highest scores were for Mental Health (mean 55.11 ± 2.25) and social aspects (mean 54.23 ± 3.07) among patients, which contributed to increasing mental health scores to the detriment of physical health. Among caregivers, the highest scores were pain (mean 63.25 ± 4.33) and mental health (mean 57.14 ± 3.17).

The worst scores were in Physical Limitation both for patients (mean 34.87 ± 4.99) and caregivers (mean 43.53 ± 6.71). The physical limitation score in caregivers contributed to reduced cumulative physical health scores compared to mental health.

It is noteworthy, in this study, that the mean values of the scores of the domains were low; practically, they did not exceed 60, even in caregivers. The pandemic was possibly responsible, and the need for improved health to improve and prolong life is highlighted [80].

The Mental Health domain (D8) assesses depression, anxiety, behavioral changes or emotional lack of control, and psychological well-being [81]. Higher patient scores were positive findings regarding cancer and the pandemic, possibly representing optimism regarding the disease, the pandemic, and the treatment.

Optimism in men was higher than in women, with a prevalence ratio of 68%, as found in other studies [82–84], which points to the greater vulnerability of women in the pandemic [73, 85].

Reduced mental health scores would be expected since individuals and their caregivers live with pain, disfigurement, dependence, isolation, side effects of treatment, and routine and even unexpected hospital admissions [47, 86].

Emotional state can influence daily activities, interfere with the ability of the individual with cancer to function, and make them feel dissatisfied with their family and social and environmental reality. This loss can perpetuate over time and lead to a functional disability with decreased autonomy and social life [87].

The mental health domain allied to the domains emotional aspects, social aspects and vitality integrate the mental component of the SF-36 instrument [88], and in the present study they demonstrated that the physical component had greater impairment than the mental one.

The pain domain (D3) includes two items ranging from 0–80. Pain influences patients' quality of life by causing suffering and disabilities, in addition to uncertainties and concerns in daily life [89].

Pain is a multifactorial symptom influenced by emotional factors and a sedentary lifestyle that can cause progressive suffering [90–92].

A recent systematic review on pain found its high prevalence in cancer. In fact, the intensity is moderate to severe in almost 40% of individuals undergoing treatment [93].

The WHO 2016 estimated that pain in almost 90% of cancer patients could be controlled with simple interventions [74]; however, many studies demonstrate that pain control in these cases is difficult. For example, in the study of 114 American oncology units with 810 individuals, 25% of the participants reported that most of the time, they lived with constant or intense pain that impacted their life and appeared to impair their quality of life [94].

Indeed, pain is associated with depression and anxiety, and negative impacts on quality of life are suggested in some studies [95–97]. Considering that the caregiver is not undergoing health treatment, the positive result obtained in this study is expected.

A study on quality of life in cancer patients in China highlighted the impact of pain on quality of life, with better quality of life associated with lower pain intensity, highlighting the importance of effective control in this population. Other studies have also demonstrated this direct influence on quality of life in Brazil [47, 98].

Cancer pain has a broad scope that requires assessing multiple aspects of the individual outcome in the physical, psychological, social, emotional, and spiritual dimensions [99–102].

Spirituality as a tool to cope with pain can manifest from practicing religion, constituting an essential component of health care [103], and in this study, religious practice was present for most participants.

The physical limitation domain (D2) is related to problems with work or daily activities due to physical health [33]. The expected result highlights these issues, considering the disease-caused limitations, the effects of treatments, the pain, and restrictions imposed by COVID-19 with social isolation. Physical limitations may include loss of function, muscle atrophy, and reduced range of motion; in addition, in chemotherapy or radiotherapy, fatigue can be one of the main consequences [47, 74], which in clinical practice is undervalued, but negatively impacts the quality of life and survival of individuals [104].

Cancer combined with medical therapies can limit the active participation of individuals with cancer in daily life activities, complicating their treatment [105]

Fatigue is a subjective feeling of physical tiredness that is disproportionate to the level of activity [106] and is a frequent symptom in chronic diseases such as cancer. It relates to the disease itself or the toxic effects of chemotherapy. Cancer patients may experience severe fatigue even after treatment ends [104].

Symptoms such as pain and fatigue affect physical and emotional function and may influence quality of life in general [47]. In the present study, function was impaired and perceived in patients mainly.

The pandemic of COVID-19 has brought current problems with uncertainties and risks, but it will also face the future problem in the face of postponements and delays in surgeries and treatments, as well as drug shortages and inadequate health care, which have contributed to social isolation and feelings of fear, loneliness, and anxiety in individuals with cancer and also in caregivers [88, 107].

Furthermore, this logjam in cancer care suggests a future overload of health systems [108]. In addition, the decrease in actions that promote early diagnosis and the need to adapt to prioritize suspected cases are reasons for concern, especially in low- and middle-income countries, since this will lead to cancer mortality in the future [109–111].

The pandemic highlighted a nationwide social rift, where changes in social routine due to the need for protective measures inevitably brought psychological, physical, social, and quality-of-life impacts perceived by the entire population. The depth of this issue was especially significant in individuals with cancer and their family nucleus, reinforcing the need to strengthen public policies that recognize and include patients and family caregivers as vulnerable.

The study had some strengths and limitations. Sending research instruments electronically was economically and logistically attractive and, even after the end of the pandemic period, deserves consideration and improvement. Another point worth noting is that the study group possibly has greater access to information and communication resources. We assumed that cancer affects the family, as it is considered a family disease; therefore, the SF-36 would be appropriate to obtain information from the patient and their caregiver. Potential limitations include that cancer stages and treatment protocols were not considered relevant, and the isolated impact of pandemic COVID-19 on participants' quality of life was not compared.

## Data Availability

Datasets generated or analyzed during the current study are not publicly available due to data privacy but are available from the corresponding author upon reasonable request.
